# USP39: a key regulator in malignant tumor progression

**DOI:** 10.3389/fonc.2025.1556011

**Published:** 2025-07-02

**Authors:** Junyan Li, Jinghua Zhong, Jianming Ye, Yi Xiang, Xiangcai Wang

**Affiliations:** ^1^ The First Clinical Medical College, Gannan Medical University, Ganzhou, Jiangxi, China; ^2^ The Oncology Department of the First Affiliated Hospital of Gannan Medical University, Ganzhou, Jiangxi, China; ^3^ Jiangxi Clinical Research Center for Cancer, Ganzhou, Jiangxi, China

**Keywords:** cancer, USP39, pre-mRNA splicing, deubiquitination, malignancy

## Abstract

Ubiquitin-specific protease 39 (USP39), a member of the USP family, plays a unique role beyond classical deubiquitination by interacting with target molecules and regulating their pre-mRNA splicing, which enhances its functional specificity compared to other USP family members. Growing evidence highlights USP39’s critical involvement in the progression of malignant tumors, where it acts as a pro-tumor factor, influencing cancer growth, proliferation, and metastasis. This paper provides a comprehensive review of the structure and functional mechanisms of USP39, emphasizing its role in regulating malignant tumor progression across various cancer types. Additionally, we explore the potential for developing targeted inhibitors based on USP39’s regulatory functions, offering a theoretical framework for future drug development. Furthermore, the study examines USP39’s contribution to resistance against antitumor therapies, highlighting its clinical relevance in advancing cancer treatment strategies. Despite the advances made, research on USP39-specific inhibitors remains limited. This work introduces a novel approach to designing inhibitors by leveraging USP39’s functional and structural characteristics, paving the way for new therapeutic avenues in cancer research.

## 1 Introduction

Cancer remains one of the most significant threats to human life and well-being, with 9.7 million people dying from it in 2022 alone. According to the International Agency for Research on Cancer (IARC), the number of cancer cases is expected to rise (1). Since the beginning of the last century, cancer treatments such as surgery, radiotherapy, immunotherapy, chemotherapy, etc. have been continuously updated, but most of these treatments are ineffective due to various adverse effects. In recent years, the existing treatments are no longer able to meet the needs of tumor treatment. Starting from the epidermal growth factor receptor (EGFR) as the first discovered tumor therapeutic target, the search for potential molecules as targeted therapeutic targets has been a hot research topic. Among the many target molecules that have been investigated, members of the USP family are considered to be among the potential biomarkers with good therapeutic prospects (2, 3).

Ubiquitin-specific proteases (USPs) are defined as regulators of protein homeostasis, maintaining protein stability by reversing ubiquitin modifications on proteins (4, 5). In malignant tumors, several USP family members interact with target proteins, modulating tumor cell growth, proliferation, migration, invasion, affecting the cell cycle, controlling apoptosis, and influencing the prognosis of cancer patients through participation in various mechanisms and signaling pathway (6, 7). For instance, in lung adenocarcinoma, the N-terminal amino acid sequence of USP38 can tightly bind to the N-terminal of KLF5, promoting tumor cell proliferation and malignant progression by maintaining the protein stability of KLF5 (8). In colorectal cancer, USP11 interacts with PPP1CA, stabilizing its protein level by removing polyubiquitination, thus activating the ERK/MAPK signaling pathway and promoting tumor progression (9). Moreover, most members of the USP family, including USP1 and USP7, participate in the regulation of tumor-associated protein networks by exerting their deubiquitinating functions. Notably, while the majority of USP family members regulate malignant tumors at the protein modification level, emerging evidence indicates that USP39 contributes to tumor progression by modulating the splicing of pre-mRNA associated with tumors during post-transcriptional modification. These findings not only highlight the diversity of tumor regulatory mechanisms mediated by USP family members, but also open new avenues for exploring tumor regulatory systems.

USP39, a member of the USP family, was initially thought to lack deubiquitination activity due to substitutions of the catalytic residues cysteine, histidine, and aspartic acid in its amino acid sequence and could only function in pre-mRNA splicing (10, 11). However, a subsequent study by Wu et al. found that USP39 stabilizes CHK2 by deubiquitinating it, demonstrating that USP39 has a deubiquitination function (12), except that it does not bind to specific proteins through the amino acid residues in the catalytic domain.USP39 is involved in various facets of human pathology and physiology, from pre-mRNA splicing and binding to specific molecules, to regulating key signaling pathways. Recent research has highlighted its crucial role in the progression of malignant tumors. With elevated expression in these cancers, USP39 promotes tumor cell growth, proliferation, invasion, migration and other malignant behaviors.

## 2 Structure of USP39

**Figure 1:**
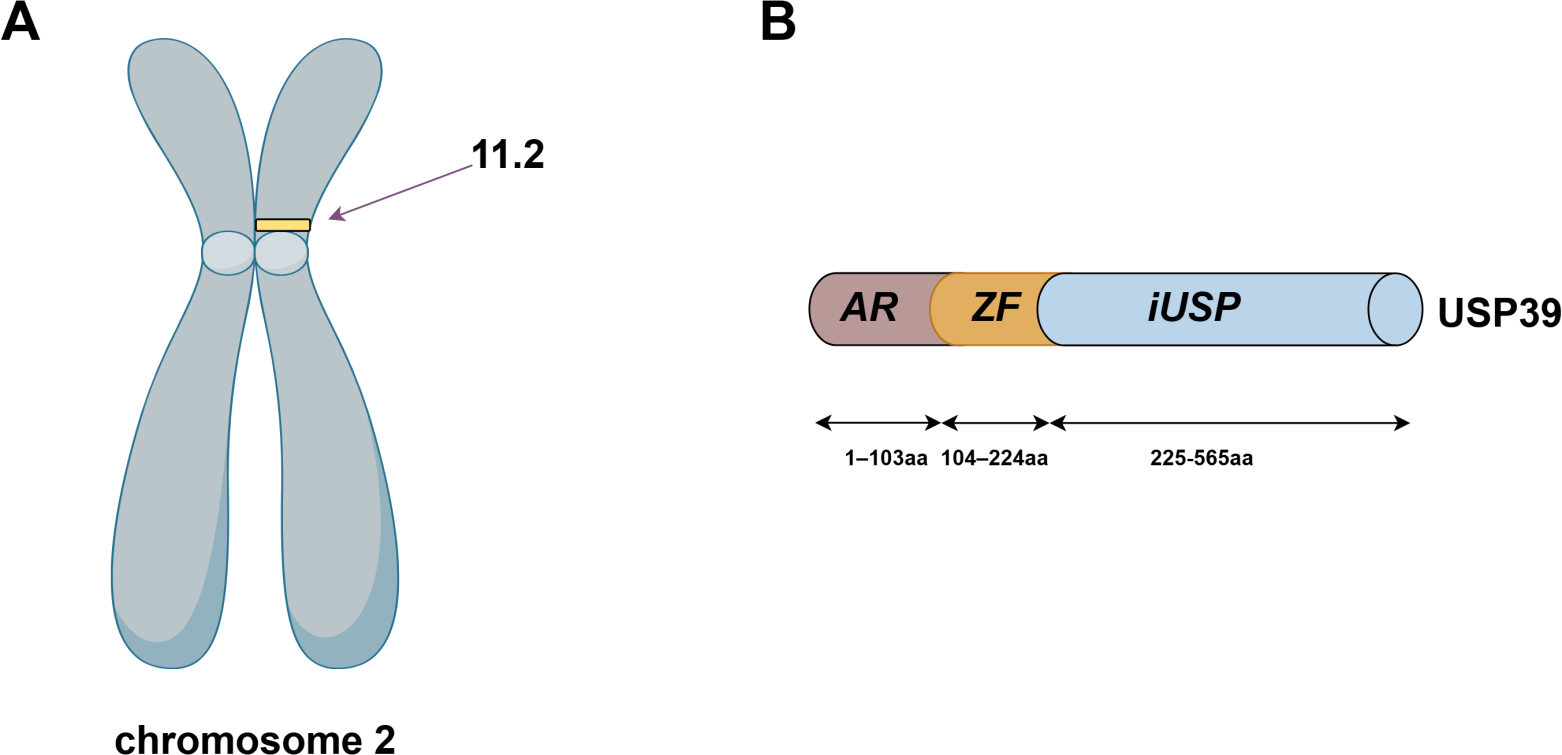
**(A)** USP39 is located on human chromosome 2p11.2. **(B)** The amino acid sequence of USP39 is 565 residues in length and consists of the AR domain (1-103aa), the ZF domain (104-224aa), and the iUSP domain (225-565aa).The figure was generated using Figdraw (https://www.figdraw.com/static/index.html#/).

**Figure 2:**
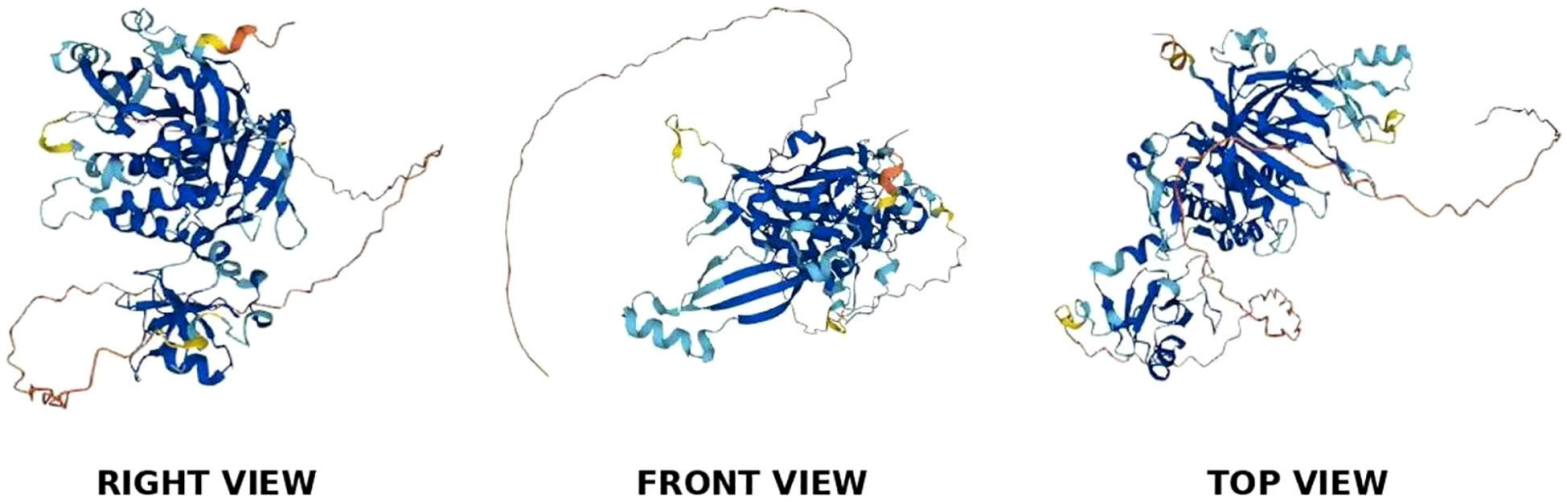
As shown in the figure, the 3D predictive model of USP39 protein, its structural composition is observed from three different angles.

**Figure 3:**
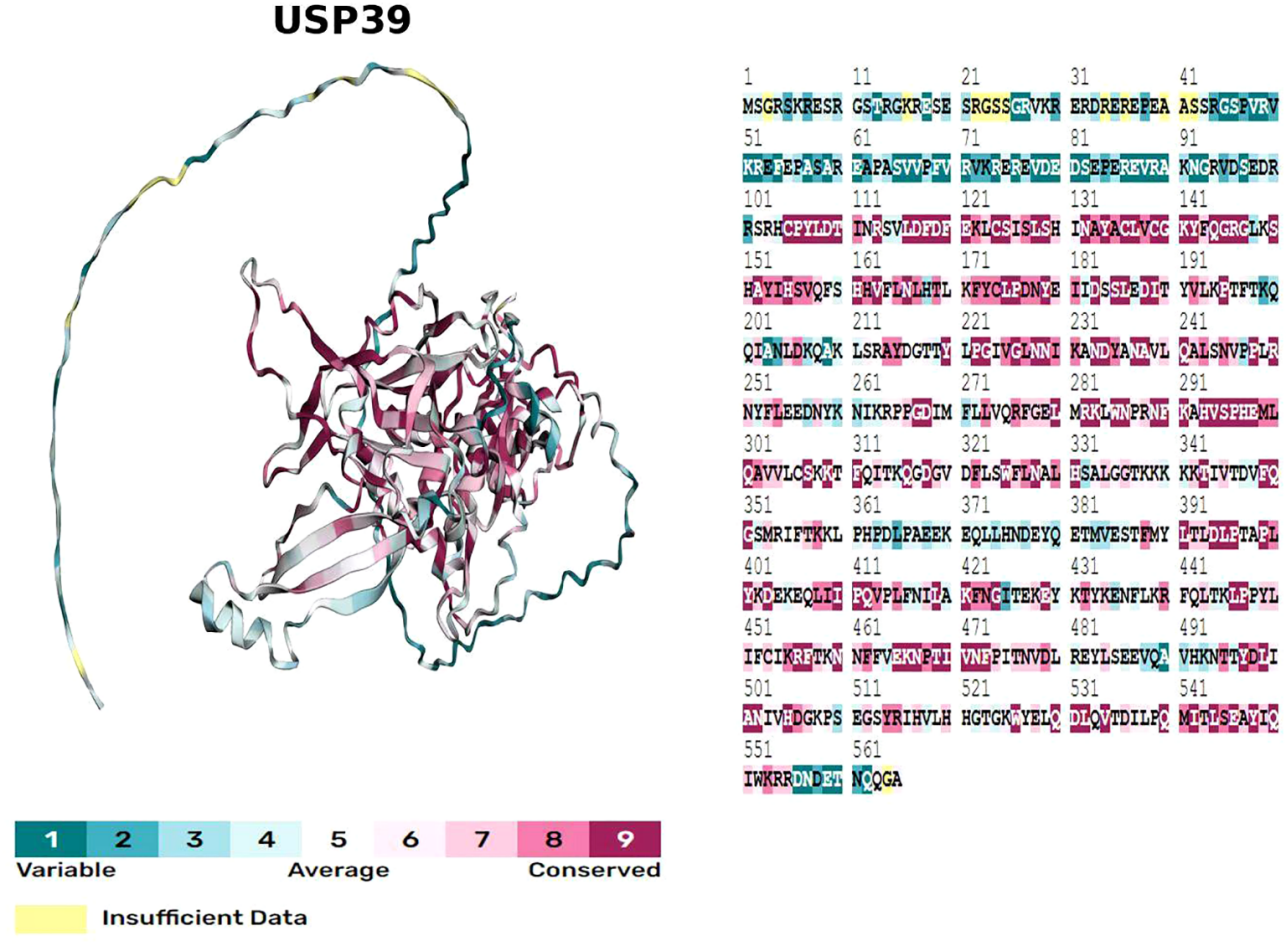
Evolutionary conservation model obtained by sequence conservation analysis of USP39 using the ConSurf server, with different colours of amino acid sites representing different levels of conservation.

The USP39 gene is located on chromosome 2, band p11.2, and is capable of producing a protein molecule with a relative molecular weight of 65 kDa after translation, consisting of 565 amino acid residues. The structural domains of USP39 include the AR domain (1-103aa), the ZF domain (104-224aa), and the iUSP domain (225-565aa) (as shown in **Figure 1**).No studies have examined the complete crystal structure of USP39, and only one study found that Sad1, the yeast homolog of USP39, may have a crystal structure similar to USP39 (13). To explore the specific structure of USP39, we chose to predict the protein model of USP39 using the AlphaFold online prediction tool, where different colors in the model represent different confidence levels (as shown in **Figure 2**), with blue and light blue representing high confidence and yellow and orange representing low confidence. Furthermore, to clarify the range of conserved and non-conserved regions of USP39, we performed sequence conservation analysis. After applying the ConSurf server analysis to obtain the results, the level of regional conservatism is classified according to the color shades, with the most variable regions represented by turquoise, while the most stable regions are represented by maroon (as shown in **Figure 3**). It is worth noting that the non-conserved region of USP39 loses the ability to bind ubiquitin due to the catalytic site mutation, but its conserved region may have functional sites for binding to specific molecules, and our structural prediction results provide some clues for exploring the potential targets of USP39 inhibitors.

## 3 Function of USP39

USP39, as a ubiquitin-specific protease, exerts multiple functional roles. Firstly, USP39 functions as part of the U4/U6. U5 tri-snRNP complex and is involved in regulating the pre-mRNA splicing of cancer-related genes. Knockdown of USP39 significantly reduces the efficiency of pre-mRNA splicing, indicating its participation in the splicing process (14). Moreover, USP39 is specifically involved in the splicing of Aurora B pre-mRNA, which plays a crucial role in regulating the cell cycle (11). Additionally, studies have revealed that USP39 can regulate the splicing of autophagy-related genes, a process essential for maintaining liver autophagy and lipid homeostasis (15). Secondly, USP39 exhibits non-canonical deubiquitination functions, specifically by binding to certain molecules and exerting regulatory effects. For example, the arginine-rich motif located in the N-terminal non-catalytic domain facilitates USP39’s binding to E proteins and stabilizes their expression by the removal of ubiquitin modifications (16). Furthermore, USP39 interacts with STAT1 and removes K6-linked ubiquitin chains, thereby stabilizing the STAT1 protein (17). Additionally, USP39 can interact with ETS2 via its N-terminal region, thereby regulating the transcriptional activity of ETS2 (18).

## 4 Role of USP39 in malignant progression of tumors

USP39 has been demonstrated to be abnormally expressed in various malignancies and is closely associated with multiple clinical characteristics (as shown in **Table 1**). *In vitro* experiments have shown that USP39 positively regulates several biological functions of tumor cells (as shown in **Table 2**). Furthermore, *in vivo* studies have confirmed that USP39 effectively modulates tumor growth (as shown in **Table 3**). This section summarizes the latest research findings on USP39 across different malignancies, elucidates its mechanisms of action, and provides a theoretical foundation for developing USP39 inhibitors.

**Table 1:** USP39 in the clinical pathological features of various tumors.

Tumor Type	Number of cases	Expression	Clinicopathological characteristics	Prognosis	Refs.
Hepatocellular Carcinoma(HCC)	374	high	tumor stage, tumor status, age, pathological stag and histologic grade.	poor	(19)
	25	high	advanced clinical grades, and overall survival (OS)	poor	(12)
	40	high	OS, DFS, vessel invasion, tumor size, and TNM stage	poor	(20)
	/	high	OS	poor	(21)
	/	high	/	/	(22)
	>100	high	pathological grade, older individuals (age ≥50)	poor	(23)
	/	high	/	poor	(24)
	606	high	age, histologic grade, pathologic stage, OS, DFS,	poor	(25)
Colorectal Cancer(CRC)	75	high	/	/	(26)
	99	high	OS	poor	(27)
Gastric Cancer (GC)	17	high	TNM stage, OS	poor	(28)
	53	high	tumor size, TNM stage, lymph-node metastasis, age, local invasion, Ki-67, OS, DFS	poor	(29)
Esophageal Squamous Cell Carcinoma(ESCC)	120	high	tumor differentiation, invasion, lymph node metastasis, and TNM stage, DFS	poor	(30)
	199	high	OS, DFS	poor	(31)
Pancreatic adenocarcinoma(PAAD)	179	high	Tumor Differentiation, TP53 Mutation Status, Immune Infiltration	poor	(32)
	90	high	TNM stage and depth of invasion	poor	(33)
Non-Small Cell Lung Cancer (NSCLC)	/	high	/	/	(34)
	30	high	/	/	(35)
Renal Cell Carcinoma(RCC)	/	high	/	poor	(36)
	176	high	TNM Stage, Fuhrman Grade, OS	poor	(37)
Prostate Carcinoma (PC)	45	high	Gleason score, PSA levels, TNM stage, and capsular invasion	poor	(38)
Breast Carcinoma(BC)	/	high	OS, DMFS, RFS, PPS	poor	(39)
	25	high	/	/	(40)
	23	high	/	/	(41)
Ovarian Carcinoma(OC)	149	high	OS, PFS	poor	(42)
	156	high	TNM Stage	poor	(43)
	119	high	chemotherapeutic resistance (carboplatin)	poor	(44)
Cervical Squamous Cell Carcinoma (CESC)	40	high	TNM Stage, nodal metastasis status.	poor	(45)
Endometrial Carcinoma (UCEC)	12	high	the clinical grade and FIGO stage of EC.	poor	(46)
Nasopharyngeal Carcinoma (NPC)	50	high	OS	poor	(47)
Head and Neck Squamous Cell Carcinoma (HNSCC)	11	high	/	/	(48)
Glioma	67	high	OS	poor	(49)
	63	high	Tumor Grade, OS	poor	(50)
Malignant Melanoma (SKCM)	70	high	tumor differentiation status	poor	(51)
Osteosarcoma (OS)	/	high	/	/	(52)

**Table 2:** The biological functions and mechanisms of USP39 in tumors.

Tumor Type	Assessed Cell Lines	Expression	Functional	Related molecule	Refs.
Hepatocellular Carcinoma(HCC)	/	/	USP39↓: Inhibit proliferation and migration.USP39↑: promote migration and migration.	/	(19)
	SK-hep-1, HepG2	Upregulated	USP39↓: Inhibit proliferation and migration. USP39↑: Promote proliferation and migration.	TRIM26	(12)
	SMMC-7721, MHCC-97H, HepG2, Hep3B, LM3	Upregulated	USP39↓: promote cell apoptosis, arrests the cell cycle, and inhibits cell proliferation USP39↑: promote proliferation Inhibit apoptosis invasion, tumor size, and TNM stage.	SP1	(20)
	Hep3B, SMMC7721, MHCC97H	Upregulated	USP39↓: Inhibit proliferation USP39↑: Promote proliferation.	SIRT7, MYST1, VHL	(21)
	SMMC-7721	Upregulated	USP39↓:Inhibit proliferation, colony formation, arrest cell cycle(Blocked in G2/M phase), Promote apoptosis USP39↑: promotes the growth of tumor cells.	FoxM1	(22)
	HepG2, SMMC-7721, BEL-7402, Huh-7, QGY-7701	Upregulated	USP39↓:Inhibit proliferation,colony formation,arrest cell cycle(Blocked in G2/M phase), Promote apoptosis USP39↑: promotes the growth of tumor cells.	p-Cdc2, p-Cdc25c, p-myt1, FoxM1	(23)
	U2OS	/	/	Aurora B	(11)
	Huh-7, Hep3B, PLC/PRF/5	/	USP39↓:Inhibit proliferation,USP39↑: Promote proliferation.	DNAAF5, PFKL	(24)
	SK-hep-1, PLC/PRF/5, and HepG2	/	USP39↓:Inhibit proliferation and migration USP39↑: rescues migration and migration.	β-catenin, TRIM26	(53)
Colorectal Cancer (CRC)	HCT116,SW620, RKO	Upregulated	USP39↓:Inhibit proliferation,Promote apoptosis, arrest cell cycle(Blocked in G2/M phase)	p21	(26)
	SW480, HT29, LoVo, Caco2	Upregulated	USP39↓:Inhibit proliferation, invasion, migration	β-catenin,TCF4, MMP2, MMP9	(27)
	RKO,HCT116,SW620	Upregulated	USP39↓:Promote apoptosis,USP39↑: Inhibit apoptosis	p53, γH2A.X	(54)
	SW1116,HCT116	Upregulated	USP39↓:Inhibit proliferation,colony formation,Promote apoptosis, arrest cell cycle(Blocked in G2/M phase)	p53, p-p53, PARP, caspase-3	(55)
Gastric Cancer (GC)	AGS, HGC27, MKN45	Upregulated	USP39↓:Inhibit proliferation,invasion, migration.USP39↑: Promote proliferation, invasion, migration	RBM39	(28)
	HGC-27, MGC-803	Upregulated	USP39↓:Inhibit proliferation, Promote apoptosis, arrest cell cycle.	miR-133a	(29)
	MGC80-3, SGC-7901, AGS	Upregulated	USP39↓:Inhibit proliferation, colony formation, arrest cell cycle(Blocked in G2/M phase)	PARP	(56)
Esophageal Squamous Cell Carcinoma(ESCC)	ECA109, KYSE30, KYSE70, TE13	Upregulated	USP39↓:Inhibit proliferatio.USP39↑: Promote proliferation	/	(30)
	KYSE30, KYSE410, KYSE450	Upregulated	USP39↓:Inhibit proliferation,invasion, migration, enhanced apoptosis induced by cisplatin (DDP).USP39↑: Promote proliferation, invasion, migration, reduced DDP-induced apoptosis	/	(31)
Pancreatic Adenocarcinoma (PAAD)	PANC-1, BxPC-3, Hs766-T, CFPAC-1, HPAF-II, MIA PaCa-2, Su86.86, SW1990, Capan-2, MPanc-96,Colo357	Upregulated	/	/	(32)
	PANC-1, AsPC-1, SW1990, BxPC-3	Upregulated	USP39↓:Inhibit proliferation,Promote apoptosis.USP39↑: Promote proliferation	miR-133a	(33)
Non-Small Cell Lung Cancer (NSCLC)	A549	Upregulated	USP39↓: Inhibit proliferation and invasion, Promote apoptosis.USP39↑: Promote proliferation and invasion and increase cell survival curve	miR-381	(34)
	95D, A549	Upregulated	USP39↓:Inhibit proliferation, arrest cell cycle (Blocked in G2/M phase), Promote apoptosis	Akt, mTOR, p53, PARP	(35)
	Calu-6, H1299, A549	Upregulated	USP39↓:Inhibit proliferation, invasion, glutamine metabolism, Promote apoptosis, arrest cell cycle	MRPL35	(57)
Renal Cell Carcinoma(RCC)	A498, OSRC-2	Upregulated	USP39↓:Inhibit proliferation, invasion, migration, Promote apoptosis, arrest cell cycle(Blocked in G2/M phase)	Akt and ERK	(36)
	786-O, ACHN, A498,769-P, Caki-1	Upregulated	USP39↓:Inhibit proliferation, arrest cell cycle(Blocked in S phase),suppressed angiogenesis in endothelial cells (HUVECs).USP39↑:increased the number of tubules and branches formed by HUVECs, promoting angiogenesis.	SRSF1,SRPK1	(37)
Prostate Carcinoma (PC)	DU145, PC-3,22RV1, LNCaP	Upregulated	USP39↓:Inhibit proliferation, promote apoptosis, arrest cell cycle (Blocked in G2/M phase)	EGFR	(38)
	PC3, LNCaP	/	USP39↓:Inhibit proliferation,USP39↑: Promote proliferation	/	(58)
Breast Carcinoma(BC)	SUM185, BT474, SUM190, BT549, MDA-MB-231, MDA-MB-453, MCF-7, ZR-75-30, MDA-MB-468, SUM159.	/	USP39↓:Inhibit proliferation, colony formation,USP39↑: Promote proliferation	FOXM1	(39)
	MDA-MB-231, HCC1937.	Upregulated	USP39↓:Inhibit proliferation, colony formation,USP39↑: Inhibit proliferation.	ax, caspase-3, Bcl-2	(40)
	MCF-7, SK-BR-3, T-47D, HCC1937, MDA-MB-231	Upregulated	USP39↓:Inhibit proliferation, colony formation, arrest cell cycle(Blocked in G0/G1 phase),Promote apoptosis	/	(41)
Ovarian Carcinoma(OC)	A2780, SKOV3, OVCAR3, OVCAR8, CAOV3, ID8	Upregulated	USP39↓:Inhibit proliferation,invasion,epithelial-mesenchymal transition (EMT).USP39↑: Promote proliferation, invasion, epithelial-mesenchymal transition (EMT)	SART1, U2AF2, PRPF3	(42)
	HO-8910, SKOV3	Upregulated	USP39↓:Inhibit proliferation and migration, arrest cell cycle(Blocked in G2/M phase).	/	(43)
	ES2 and SKOV3	Upregulated	USP39↓:Inhibit proliferation, colony formation,invasion,migration,arrest cell cycle(Blocked in G2/M phase),Increased sensitivity to carboplatin-induced apoptosis,USP39↑: Promote proliferation, colony formation, invasion, migration, Enhanced chemoresistance to carboplatin.	EGFR, cyclin B1, cleaved caspase-3, cleaved PARP	(44)
Cervical Squamous Cell Carcinoma (CESC)	CaSki, SiHa, C-33A	Upregulated	USP39↓:Inhibit proliferation, colony formation, Promote apoptosis,USP39↑:Promote proliferation.	SIRT7,FOXM1	(45)
Endometrial Carcinoma (UCEC)	Ishikawa, KLE, RL95-2, HEC-1A, HEC-1B	Upregulated	USP39↓:Inhibit proliferation,migration,arrest cell cycle(Blocked in G0/G1 phase),Promote apoptosis,USP39↑: Promote proliferation, migration	PGK1	(46)
Nasopharyngeal Carcinoma (NPC)	SUNE-1, CNE-1, HNE-1, CNE-2, C666-1, and HONE-1	/	USP39↓:Inhibit proliferation, colony formation, USP39↑: Promote proliferation, colony formation	miR-26b-3p	(47)
Head and Neck Squamous Cell Carcinoma (HNSCC)	CAL27 and SCC25	/	USP39↓:Inhibit proliferation and invasion	STAT1	(48)
Glioma	U251 and U87	Upregulated	USP39↓:Inhibit proliferation, colony formation, arrest cell cycle(Blocked in S and G2/M phase),Promote apoptosi.USP39↑: Promote proliferation.	Cyclin B1	(49)
	U251 and U87	/	USP39↓:Inhibit migration and invasion.USP39↑: Promote migration and invasion	ADAM9, Integrin β1	(50)
Malignant Melanoma (SKCM)	A375, M14	/	USP39↓:Inhibit proliferation, Promote apoptosis, arrest cell cycle(Blocked in G0/G1 phase)	ERK1/2	(51)
Osteosarcoma (OS)	U2OS, SW1353, Saos-2	Upregulated	USP39↓:Inhibit proliferation, colony formation, Promote apoptosis, arrested cell cycle at G2/M phase	p21, Cyclin A2, Caspase-3, PARP	(52)
Leukemia	Jurkat, HL-60, K-562	Upregulated	USP39↓:Inhibit proliferation, promote apoptosis, arrest cell cycle (Blocked in G2/M phase)	IRF1, Caspase 8, SP1	(59)

The symbol "↑" signifies upregulation of USP39 expression, while "↓" indicates downregulation of USP39 expression.

**Table 3:** Effects of USP39 on growth and metastasis of cancer xenografts.

Disease type	Animal models	Results	Refs.
HCC	6–8-week-old male nude mice	USP39↓:tumor volume and weight↓	(12)
	5-week-old nude mice	USP39↓:tumor volume and weight↓	(20)
	6-week-old nude mice	USP39↓:tumor volume and weight↓ USP39↑:tumor volume and weight↑	(21)
	4–6 weeks-old-female BALB/c nude mice	USP39↓:tumor volume and weight↓	(22)
	4–6 weeks-old BALB/c nude mice	USP39↓:tumor volume and weight↓	(23)
NPC	5 weeks-old-male BALB/c nude mice	USP39↓:tumor volume and weight↓	(47)
Glioma	6–8 weeks-old-male BALB/c nude mice	USP39↓:tumor volume and weight↓ USP39↑:tumor volume and weight↑	(49)
	6–8 weeks-old-male BALB/c nude mice	USP39↓:reduced tumor area and increased survival USP39↑:developed tumor area and reduced survival	(50)
OC	4–5 weeks-old-female BALB/c nude mice	USP39↓:tumor volume and weight↓ USP39↑:tumor volume and weight↑	(42)
	4–6 weeks-old-female BALB/c nude mice	USP39↓:tumor volume and weight↓	(43)
	5–6 weeks-old-female BALB/c nude mice	USP39↓:tumor volume and weight↓ USP39↑:tumor volume and weight↑	(44)
BC	5 weeks-old-female BALB/c nude mice	USP39↓:tumor volume and weight↓	(39)
CRC	6–8 weeks-old-male BALB/c nude mice	USP39↓:tumor volume and weight↓	(54)
	4–6 weeks-old-male BALB/c nude mice	USP39↓:tumor volume and weight↓ USP39↑:tumor volume and weight↑	(26)
ESCC	3–4 weeks-old-male BALB/c nude mice	USP39↓:tumor volume and weight↓	(30)
	6 weeks-old-male BALB/c nude mice	USP39↓:tumor volume and weight↓ USP39↑:tumor volume and weight↑	(31)
PAAD	4–6 weeks-old-male BALB/c nude mice	USP39↓:tumor volume and weight↓ USP39↑:tumor volume and weight↑	(33)
SKCM	5-week-old male BALB/c nude mice	USP39↓:tumor volume and weight↓	(51)

The symbol "↑" signifies upregulation of USP39 expression, while "↓" indicates downregulation of USP39 expression.

### 4.1 Hepatocellular carcinoma

A large number of studies have found that USP39 is highly expressed in hepatocellular carcinoma tissues and cells, and the aberrant expression of USP39 may significantly contribute to the development of hepatocellular carcinoma. In addition, Ni et al. found that the overexpression of USP39 was closely related to histological grading, pathological stages and other clinicopathological features by analyzing the cBioPortal and TCGA databases (25). Liao et al. discovered through gene enrichment analysis that USP39 regulates key signaling pathways, including the cell cycle, DNA replication, and mismatch repair (19).

USP39 plays a critical role in regulating the malignant progression of HCC by binding to and interacting with various protein molecules. Dong et al. demonstrated that USP39 promotes the proliferation of HCC cells by interacting with the SP1 protein and stabilizing it through deubiquitination (20). Moreover, Dong et al.’s study revealed that USP39 interacts with SIRT7, which deacetylates USP39 to enhance its stability, thereby further promoting HCC development (21). Additionally, Xiaomei Li et al. discovered that USP39 co-regulates ZEB1 expression in conjunction with TRIM26, though the two exhibit opposing effects. TRIM26 reduces ZEB1 stability through ubiquitination, thereby suppressing ZEB1 expression. In contrast, USP39 reverses this process by stabilizing ZEB1 via deubiquitination, ultimately driving HCC progression (12). Tumor glycolysis, a hallmark of the tumor microenvironment, not only provides energy to support tumor growth but also facilitates malignant progression by producing lactic acid (60, 61). Liu et al. found that USP39 can be recruited by DNAAF5 to interact with and stabilize PFKL, effectively promoting aberrant glycolysis within the HCC tumor microenvironment (24).

USP39 may also function in regulating pre-mRNA splicing in HCC. Aurora B maintains accurate chromosome segregation by correcting erroneous attachments between the spindle and kinetochores. Van Leuken et al. found that knocking down USP39 expression effectively inhibits the splicing of Aurora B pre-mRNA, leading to cell cycle arrest in HCC cells and suppressing their growth and proliferation (11). Similar mechanisms have been observed in regulating FoxM1 pre-mRNA splicing, resulting in G2/M cell cycle transition blockade (22, 23). β-catenin serves as a critical regulator within the Wnt/β-catenin signaling pathway, and its abnormal expression has been linked to the onset and progression of various types of tumors. Wang et al. found that USP39 not only promotes the deubiquitination of β-catenin by interacting with β-catenin, but also is able to indirectly promote the deubiquitination of β-catenin through regulating pre-mRNA splicing of TRIM26, thereby promoting the proliferation and migration of HCC (53). Unlike other USP family members, USP39, as an important component of the spliceosome, demonstrated its unique pre-mRNA splicing function.

USP39 shows modulation of multiple biological functions of malignant tumors in HCC, giving it potential as a pro-tumorigenic factor.

### 4.2 Colorectal cancer

USP39 is highly overexpressed in colorectal cancer tissues and cells, while USP39 knockdown suppresses all tumor cell functions. In addition, using Kaplan-Meier analysis, the researchers discovered that elevated USP39 expression was associated with poor overall survival in cancer patients, indicating that USP39 might contribute to the malignant progression of colorectal cancer.

In colorectal cancer, USP39 regulates apoptosis and cell proliferation via key molecular pathways. Xing et al. found that USP39 knockdown accelerated apoptosis by increasing the levels of apoptosis-related proteins, including PARP, p53, and caspase-3 (55). In addition, Yuan et al. found that USP39 could activate the Wnt/β-catenin signaling pathway by modulating critical proteins involved in the pathway, such as β-catenin, TCF4, MMP2, and MMP9, thereby facilitating the growth and proliferation of colorectal cancer cells (27). Moreover, P21 serves as a transcriptional target of the p53 tumor suppressor, and USP39 is able to regulate the stability of P21 by modulating the half-life and promoter activity of P21 in the p53/p21/CDC2/cyclin B1 axis, which in turn regulates colorectal cancer development (26).

Cisplatin is usually combined with other cytotoxic drugs in the treatment of colorectal cancer, but the problem of resistance to cisplatin has led to unsatisfactory patient outcomes. Yuan et al. found that knocking down USP39 expression in a cisplatin-treated cell line indirectly enhances the cisplatin-induced apoptosis of colorectal cancer cells by increasing the stability of the p53 protein (54), suggesting that USP39 may play a role in the epigenetic regulation of cisplatin resistance. It is important to highlight that USP39 serves as a tumor-promoting factor in the majority of malignant cancers, and the combination of cisplatin and USP39 inhibitors may effectively improve the sensitivity of patients to the drug and enhance the therapeutic effect. Therefore, the development of USP39 inhibitors may be of high clinical significance. These findings suggest that combining USP39 inhibitors with cisplatin could improve therapeutic outcomes, underscoring USP39’s potential as a drug target.

### 4.3 Gastric cancer

USP39 shows high levels of expression in gastric cancer (GC) tissues and cells, while its knockdown notably suppresses cell proliferation and colony formation, and overexpression of USP39 reversed the above results (56), suggesting that USP39 might function as a tumor-promoting factor in gastric cancer.

In GC, USP39 has been found to interact with several molecules. PARP is an enzyme widely found in eukaryotic cells, which is closely related to tumor development, and PARP inhibitors are currently used in tumor chemotherapy. Wang et al. found that USP39 can interact with PARP protein, and by knocking down USP39, the cleavage of amino acid 214 of PARP protein was increased, resulting in the loss of PARP protein function and promoting apoptosis in gastric cancer cells (56). RBM39 is a protein with multiple functions in cancer development, involved in transcriptional regulation and pre-mRNA selective splicing. Lu et al. found that USP39 can interact with RBM39 and stabilize it through removal of the polyubiquitin chain by K48 deubiquitination, therefore promoting tumor progression (29). miR-133a,recognized as a highly conserved non-coding RNA, has been reported to exhibit tumor-suppressive functions in multiple malignancies (62–67). Dong et al. found that miR-133a directly targets USP39, suppressing its protein expression through negative regulation and effectively hindering the progression of gastric cancer (28). Notably, the inhibitory effect of miR-133a on USP39 suggests that miR-133a has the potential to act as a USP39-specific inhibitor. However, further cell culture and animal model experiments are needed to validate this hypothesis.

USP39’s role in gastric cancer, which involves regulating tumor progression through interactions with PARP, RBM39, and miR-133a, emphasizes its potential as a promising clinical target for therapeutic applications.

### 4.4 Esophageal squamous cell carcinoma

USP39 is upregulated in esophageal squamous cell carcinoma (ESCC) tissues, with high expression negatively associated with patient prognosis. In further mechanistic studies, Zhao et al. found a link between USP39 and the activation of the mTOR signaling pathway.USP39 promoted the malignant progression of ESCC by enhancing the splicing and maturation of Rictor pre-mRNA and promoting the activity of mTORC2 (30). Interestingly, in the study by Zhu et al., the researchers found through flow cytometry analysis that overexpression of USP39 in tumor cells that were not treated with DDP did not affect the apoptosis rate. However, in tumor cells treated with DDP, overexpression of USP39 was able to reduce the apoptosis rate (31), suggesting that USP39 may influence the effect of cisplatin through a certain regulatory mechanism, which requires further research to clarify.

### 4.5 Pancreatic cancer

Pancreatic cancer is considered the most malignant tumor due to its insidious onset and rapid progression, and its mortality rate is still increasing (68, 69). USP39 has been found to be aberrantly expressed in pancreatic cancer tissues and cells, and regulates tumorigenesis and development by participating in a variety of biological processes. Cai et al. found that USP39 can act as a direct target of miR-133a (33). miR-133a has previously been shown to target USP39 in gastric cancer, suggesting that there may be specific binding between USP39 and miR-133a in a variety of malignant tumors. In addition, Wang et al. found that, through bioinformatics analysis, elevated USP39 expression was predictive of poor prognosis in patients with pancreatic ductal adenocarcinoma and was closely linked to various clinicopathological features, including tumor differentiation and immune infiltration levels (32).

The above study demonstrated that USP39 is involved in the regulatory network of malignant progression of pancreatic cancer, and the phenomenon of inhibiting tumor progression by suppressing the expression of USP39 suggests that USP39 may be a potential therapeutic target for pancreatic cancer.

### 4.6 Tumors of the respiratory tract

#### 4.6.1 Lung cancer

USP39 was aberrantly expressed in tissues and cells of several lung cancer types compared to normal lung tissue. In addition, researchers found that the proliferative activity of tumor cells was inhibited in 95D and A549 cell lines with knockdown of USP39 (35), and these findings indicate that USP39 could contribute to the promotion of tumor growth in lung cancer.

Non-small cell lung cancer (NSCLC), which encompasses large cell lung cancer, adenocarcinoma, squamous cell carcinoma, and other subtypes, is the predominant pathological subtype of lung cancer, accounting for approximately 85% of all lung cancers. miR-381, a microRNA (miRNA), is capable of influencing the malignant progression of tumors by regulating specific genes and signaling pathways. In a study of lung adenocarcinoma, Hou et al. found that miR-381 was able to inhibit tumor proliferation and invasion by targeting and regulating the expression of LMO3 (70), suggesting that miR-381 may be a tumor suppressor. Interestingly, miR-381 was able to bind to a site on the USP39 3′UTR sequence in NSCLC and was equally effective in inhibiting cancer cell development by suppressing USP39 expression (34). The above studies demonstrate the multiple regulatory pathways by which miR-381 inhibits tumors, as well as the efficacy and necessity of inhibiting this particular target of USP39.

In the tumor microenvironment, glutamine metabolism is an important regulatory mechanism to provide energy for tumors, and SLC7A5 and MRPL35 are important regulators of this mechanism. Hou et al. found that USP39 can bind to MRPL35 to exert a deubiquitylation effect and increase the overexpression of MRPL35 by preventing it from being degraded by the ubiquitin-proteasome pathway, which then promotes glutamine metabolism and tumor progression (57).The regulation of glutamine metabolism by USP39 suggests that USP39 may indirectly affect tumor development by regulating the tumor microenvironment, reflecting the diversity of mechanisms of tumor regulation by USP39.

### 4.7 Tumors of the urinary tract

#### 4.7.1 Renal cell carcinoma

USP39 exhibits elevated expression in renal cell carcinoma tumor tissues and cells compared to normal kidney tissues, and patients with higher USP39 levels experience significantly reduced overall survival (OS).

USP39 can affect the development of renal cell carcinoma by activating multiple signaling pathways and indirectly regulating the variable splicing of VEGF-A pre-mRNA. After constructing USP39 knockdown cell lines, Xu et al. observed that the proliferation, invasion, migration and other functions of tumour cells were reduced, and further showed that the phosphorylation levels of Akt and ERK, key regulators of PI3K/Akt and MAPK/ERK signaling pathways, were also significantly reduced by Western blot, suggesting that USP39 may activate the relevant signaling pathways by regulating the phosphorylation levels of Akt and ERK (36). VEGF-A is an important regulator of angiogenesis and promotes tumor growth by increasing angiogenesis (71). Pan et al. showed that the ZnF (zinc finger) structural domain and the UCH1/UCH2 structural domains of USP39 were able to bind to the action site on SRSF1, driving VEGF-A pre-mRNA splicing through enhanced phosphorylation of SRSF1, thereby generating as many VEGF-A165a isoforms as possible to activate the angiogenic signalling pathway and promote tumour growth (37).

The above studies demonstrated that USP39 may play multiple regulatory roles in renal cell carcinoma, but further studies are needed to determine whether USP39 cross-regulates the development of renal cell carcinoma in other tumor-related signaling pathways.

#### 4.7.2 Prostate cancer

USP39 is aberrantly expressed in prostate cancer tissues and cells and plays a role as a pro-tumorigenic factor in the malignant progression of prostate cancer. In addition, overexpression of USP39 was strongly associated with Gleason score, biochemical recurrence (BCR) and disease-free survival (DFS).

EGFR, a transmembrane tyrosine kinase, can activate various intracellular signaling pathways, and its activation or mutation has been demonstrated to promote tumorigenesis in numerous malignancies (72, 73). Huang et al. found that USP39 was able to stabilise the 3′-UTR region of EGFR mRNA by regulating EGFR pre-mRNA splicing, and the 3′-UTR region is important for post-transcriptional regulation (38). Stable expression of EGFR protein levels effectively promotes malignant progression of prostate cancer by activating multiple signaling pathways. Currently, targeted therapy against EGFR is the key to tumor treatment, and the development of various EGFR inhibitors such as gefitinib and erlotinib in the past has significantly changed the therapeutic strategy of many cancers. The effective regulation of EGFR pre-mRNA splicing function by USP39 suggests that it may have the potential to become a target for targeted therapy of prostate cancer.

SUMO is a ubiquitin-like protein that can alter the structure, localization, and increase the stability of target proteins by binding to them. Wen et al. found that there are multiple SUMO binding sites in the RS-like domain of USP39. It is noteworthy that prostate cancer cell proliferation is inhibited when SUMO binds to sites on USP39, but the attraction phenomenon can be reversed when these SUMO binding sites are mutated (58), suggesting that SUMO modification can inhibit the tumor-promoting effects of USP39. The above studies suggest that USP39 expression can be effectively inhibited by binding to specific targets on the RS-like domain of USP39, but further clarification of which compounds can bind to these sites will require extensive experimental characterization in the future.

### 4.8 Gynecological tumors

#### 4.8.1 Breast cancer

Immunohistochemical staining analysis revealed that USP39 expression was elevated in breast cancer cells. In the USP39 knockdown MCF-7 cell line, breast cancer cell proliferation was inhibited, and the cell cycle transition from G0 to G1 was blocked (41). The above experiments suggest that USP39 may play a role as a pro-tumorigenic factor in the malignant progression of breast cancer.

FoxM1, a transcription factor that regulates several biological processes, has been shown to play a key role in tumorigenesis, drug resistance, and epithelial-mesenchymal transition (EMT). FoxM1 is aberrantly expressed in cells of several breast cancer subtypes and can affect cell cycle progression by modulating cell cycle-related genes (74–76). Triple-negative breast cancer is the most malignant subtype of breast cancer with negative expression of estrogen receptor (ER), progesterone receptor (PR), and human epidermal growth factor receptor 2 (HER2), and is associated with poor therapeutic response and prognosis. Studies revealed that FoxM1 and USP39 were both overexpressed in triple-negative breast cancer cells. The abnormal expression of FoxM1 enhanced the expression of genes associated with centrosome amplification and aggregation, which promoted tumor progression by inducing an imbalance in cell cycle regulation (77), whereas USP39 could not only indirectly affect cell cycle progression by deubiquitylating FoxM1 (39), but also affect tumor cell apoptosis by regulating the balance between the expression levels of the pro-apoptotic factor Bax and the anti-apoptotic factor Caspase-3 (40). Notably, the regulation of FoxM1 expression by USP39 has only been confirmed in cell lines of triple-negative and estrogen receptor (ER)-positive breast cancer subtypes. Whether this regulatory mechanism exists in other breast cancer subtypes remains unclear and requires further experimental validation.

In conclusion, the function of USP39 in influencing the progression of malignant tumors by regulating the expression of various molecules makes it a promising potential target for breast cancer therapy.

#### 4.8.2 Ovarian cancer

USP39 is overexpressed in ovarian cancer tissues and is closely linked to TNM staging. *In vivo* and *in vitro* assays have demonstrated that USP39 knockdown suppresses tumor growth, inhibits cell proliferation, and blocks the G2-to-M phase transition of the cell cycle. EMT is a key link in tumor metastasis, and Yan et al. found that USP39 is able to regulate EMT by activating the p53/p21 signaling pathway to promote tumor cell metastasis (43). HMGA2, as a non-histone chromatin structure transcription factor, is upregulated in expression in almost all human malignancies (78, 79). Recently, Wang et al. found that USP39 enhances HMGA2 expression in high-grade serous ovarian cancer (HGSOC) by facilitating the efficient splicing of HMGA2, thereby indirectly contributing to tumor progression (42).

Increased tumor resistance to chemotherapeutic agents usually leads to malignant progression in tumor patients. Cisplatin is a common chemotherapeutic agent used to treat ovarian cancer patients. In an animal model constructed by Wang et al, it was found that USP39 overexpression reduced the sensitivity of ES2 cell line cells to cisplatin, whereas this effect was reversed following USP39 knockdown (44), indicating that USP39 expression levels influence cellular sensitivity to cisplatin, similar to the fact that knockdown of USP39 improves the sensitivity of gastric cancer cells to carboplatin.

#### 4.8.3 Cervical squamous cell carcinoma

USP39 expression was upregulated in CSCC tissues and in several cell lines. SIRT7, a deacetylase implicated in a variety of malignancies, has been found to play a pro-tumorigenic role in hepatocellular carcinoma, and notably, SIRT7 expression was also upregulated in CSCC. The study by Yu et al. demonstrated by immunoprecipitation assay and deacetylation assay that SIRT7 not only improves the stability of USP39 protein by deacetylating the K428 site on the USP39 structure, but also participates in the activation of the SIRT7/USP39/FOXM1 positive feedback loop, providing a new idea for targeted treatment of CSCC (45).

#### 4.8.4 Endometrial cancer

In endometrial cancer, USP39 is involved in a variety of mechanisms and pathways, not only interacting with PGK1 to improve the stability of PGK1, but also regulating the process of glycolysis by activating the PI3K/AKT/HIF-1α signaling pathway, and these modifications can effectively promote the growth and proliferation of tumor cells. Histone lactonization, as a protein modification, has been shown to play a role in tumorigenesis. In endometrial cancer cells, histone lactonization can stimulate USP39 overexpression by enriching H3K18la in the USP39 promoter region, thereby promoting the growth and proliferation of endometrial cancer cells (46).

### 4.9 Head and neck cancers

#### 4.9.1 Nasopharyngeal carcinoma

LINC00520, a highly conserved long non-coding RNA (lncRNA), plays a role in regulating malignant tumor progression by interacting with various miRNAs (80). Interestingly, its expression level was found to be upregulated in most malignant tumors, but in cutaneous squamous cell carcinoma, LINC00520 expression was instead downregulated (81), suggesting that LINC00520 may have a dual regulatory role in tumors. In contrast, in nasopharyngeal carcinoma, analysis by quantitative real-time fluorescence PCR (qRT-PCR) technology showed that LINC00520 was aberrantly expressed in tissues, and further analysis of clinical data indicated that LINC00520 may be associated with poor prognosis. Notably, LINC00520 can indirectly regulate the expression of USP39 in the LINC00520/miR-26b-3p/USP39 pathway model constructed by Xie et al. LINC00520 affects the process of malignant progression of nasopharyngeal carcinoma by antagonizing the expression level of USP39 regulated by miR-26b-3p (47), suggesting that USP39 may play a role as a regulator in nasopharyngeal carcinoma.

#### 4.9.2 Head and neck squamous cell carcinoma

LC-MS/MS analysis showed that USP39 was expressed at higher levels in head and neck squamous cell carcinoma (HNSCC) tissues than in adjacent normal tissues. STAT1 is a transcription factor that plays a pivotal role in regulating tumor progression and controls gene expression by activating the JAK-STAT signaling pathway. In HNSCC, both USP39 and STAT1 expression levels were upregulated. Hu et al. found that knockdown of either USP39 or STAT1 in CAL27 and SCC25 cell lines inhibited cell growth and proliferation, suggesting that both USP39 and STAT1 may play a tumor-promoting role in HNSCC. Meanwhile, it was also found that knockdown of USP39 alone inhibited the expression of STAT1 (48), suggesting that USP39 may affect tumor cell development by regulating STAT1, but further mechanistic studies are needed for further investigation.

#### 4.9.3 Glioma

The expression levels of USP39 are elevated in glioma tissues and cells. In addition, *in vitro* experiments exploring the functional role, knockdown of USP39 revealed inhibition of glioma cell growth and proliferation.

USP39 interacts with multiple protein molecules in gliomas. Xiao et al. found that USP39 not only interacts with and stabilizes cyclin B1 by deubiquitinating it, but also stabilizes the expression of ADAM9 by promoting the maturation of ADAM9 mRNA, and that these regulatory roles of USP39 promote the malignant growth of glioma cells (49, 50). Interestingly, USP39 acts as a pro-tumorigenic factor in gliomas that can also be regulated by other molecules. In the circCLSPN-miR-370-3p-USP39 signaling network constructed by Hu et al, circCLSPN was found to be able to influence the expression level of USP39 through activation of this network, thereby regulating glioma development (82).

The aforementioned research underscores the critical function of USP39 regulation in glioma and provides a strong basis for further investigation into its specific mechanisms.

#### 4.9.4 Medullary thyroid cancer

Medullary thyroid carcinoma is a neuroendocrine tumor, and the presence of amyloid deposits in the pathological tumor mesenchyme is its unique feature. Its main treatment modalities include surgery, radiotherapy, and systemic therapy (83, 84). An et al. found that most cells in USP39-knockdown TT cell lines were arrested in the G2/M phase, leading to inhibited cell proliferation. In addition, Western blot analysis showed that the expression of cell cycle-related proteins Cyclin B1 and CDK1 was downregulated when USP39 was knocked down (85). These findings suggest that USP39 might influence tumor cell proliferation through the regulation of molecules and proteins associated with the cell cycle. However, the specific function and underlying mechanism of USP39 remain uncertain and warrant further exploration.

#### 4.9.5 Oral squamous cell carcinoma

In CAL27 cell lines, USP39 knockdown suppressed cell proliferation, and flow cytometry analysis revealed that the majority of tumor cells were arrested in the S and G1/M phases of the cell cycle following USP39 knockdown, indicating that USP39 might influence the malignant progression of tumor cells through the regulation of cell cycle-related protein molecules. Caspase 3 is an apoptosis-associated protease and PARP is a DNA repair enzyme. PARP is cleaved by caspase 3 during apoptosis, and the activation status of both is an important biomarker for assessing the occurrence of apoptosis. Western blot analysis showed that when USP39 was knocked down, caspase 3 and PARP were activated, leading to apoptosis in tumor cells (86), demonstrating that USP39 can indirectly affect tumor development by regulating the expression levels of key apoptotic proteins.

#### 4.9.6 Neuroblastoma

In a related study, genes associated with prognosis were screened by pathway enrichment analysis and an optimized Cox model for NB risk stratification was constructed. The analysis showed that USP39 expression levels were increased among patients with poor prognosis relative to individuals with good prognosis. Additionally, single-cell RNA sequencing demonstrated that USP39 plays a role in regulating RNA splicing in neuroblastoma (87).

### 4.10 Other malignancies

#### 4.10.1 Melanoma

Immunohistochemical staining of clinical samples revealed that USP39 expression was markedly elevated in melanoma tissues compared to benign nevus tissues. In addition, the study also found that USP39 expression was elevated in poorly differentiated melanoma tissues, indicating a correlation between USP39 and the degree of tumor differentiation. In the A375 and M14 cell lines constructed by knocking down USP39, the researchers observed that melanoma cell proliferation was suppressed, and cell cycle progression was arrested at the G0/G1 phase, while the growth of tumors in the nude mice transplantation tumor model was also inhibited *in vivo*, suggesting that the inhibition of USP39 expression can alleviate the malignant progression of melanoma to some extent. ERK1/2 are key regulatory proteins in the MAPK signaling pathway, with their abnormal activation being shown to regulate tumor development (88, 89). Zhao et al. found that USP39 activates the MAPK signaling pathway by inhibiting the phosphorylation modification of ERK1/2, which promotes tumor progression (51), suggesting that the regulation of cellular function by USP39 in melanoma may function by affecting key molecules of the signaling pathway.

The role of USP39 in indirectly affecting tumor progression by regulating key proteins of the MAPK pathway provides a new means and direction for the treatment of poorly differentiated refractory melanoma in the clinic, and targeted inhibitors of USP39 are worthy of further investigation.

#### 4.10.2 Osteosarcoma

USP39 expression is upregulated in osteosarcoma, and *in vitro* studies revealed that silencing USP39 suppressed osteosarcoma cell growth and proliferation, inducing cell cycle arrest at the G2/M phase in the majority of cells. Furthermore, Gan et al. analyzed the effects of USP39 knockdown on osteosarcoma cells and, through Western blot analysis, observed an increase in the cleavage of poly-PARP and caspase-3, indicating that the apoptotic program was activated, thereby promoting apoptosis in osteosarcoma cells (52).

The above experiments demonstrated the abnormal expression level of USP39 in osteosarcoma and the pro-tumorigenic function it exerts, but further exploration of the regulatory mechanism of USP39 involvement is still lacking.

#### 4.10.3 Leukemia

USP39 was highly expressed in leukemia cells, and evidence from the TCGA database indicated that its overexpression is linked to poor prognosis. In the USP39 knockdown cell line established by Liu et al., leukemia cell proliferation and invasion were suppressed. Flow cytometry analysis showed that the cell cycle was arrested in the G2/M phase, and apoptosis was promoted, indicating that USP39 contributes to the growth and proliferation of leukemia cells. Furthermore, through microarray analysis in bioinformatics, the researchers found that USP39 was correlated with target genes such as IRF1, caspase 8 and SP1 (59), which play key regulatory roles in regulating tumor apoptosis and proliferation, suggesting that USP39 can influence tumor growth in leukemia cells by participating in various signaling pathways.

The above studies have confirmed the tumor-promoting function of USP39 in leukemia, but the specific molecules that USP39 interacts with and the pathways that USP39 activates to play its role are still unknown and require further in-depth studies in the future.

## 5 Other diseases

Acute myocardial infarction (AMI) is a serious complication of coronary atherosclerosis, and its prevalence and mortality have been increasing in recent years. Studies have identified USP39 as a key regulator in the pathology of AMI. circUSP39, a cyclic RNA molecule of the USP39 gene, is upregulated in AMI. Knockdown of USP39 can effectively inhibit oxidative stress, inflammation and apoptosis in cardiomyocytes and ameliorate cardiac damage caused by acute myocardial infarction by regulating the USP39/miR-362-3p/TRAF3 axis, resulting in the downregulation of TRAF3 (90). In addition, knockdown of USP39 was also found to activate the USP39/miR-499b-5p/ACL1 axis, which further inhibited ACSL1 expression by negatively regulating miR-499b-5p expression, effectively alleviating cardiomyocyte damage under hypoxic conditions (91).

USP39 has also been found to be involved in the inflammatory response process. Knockdown of USP39 in macrophages increases the expression and secretion of pro-inflammatory cytokines, causing an inflammatory response. IκBα has been shown to be a negative regulator in the inflammatory response to NF-κB, and USP39 binds to IκBα preventing IκBα degradation by removing the ubiquitin chain of IκBα at Lys48-linked ubiquitination to inhibit inflammation occurrence (92).

Vascular remodeling is a major risk factor contributing to the progression and aggravation of cardiovascular diseases, with USP39 identified as playing a role in regulating this process. The proliferation and migration of vascular smooth muscle cells (VSMC) is one of the main processes of vascular remodeling. Knockdown of USP39 expression was found to suppress VSMC migration, arrest the cell cycle, and inhibit VSMC proliferation by downregulating Cyclin D1 and CDK4 expression, ultimately achieving the goal of impeding the process of vascular remodeling. The regulatory role of USP39 in vascular injury makes it a potential therapeutic target for inhibiting vascular remodeling, and further studies on the specific pathways in which USP39 plays a regulatory role are needed in the future (93).

USP39 also plays a role in DNA damage repair, as USP39 stabilizes CHK2 by deubiquitinating CHK2, which in turn promotes DNA repair by phosphorylating downstream factors (94), and USP39 is also able to influence DNA repair by regulating NHEJ at DNA damage sites (13).

In summary, USP39 plays a critical regulatory role in malignant tumors as well as in various physiological and pathological processes, underscoring its significance as an essential regulatory molecule in the human body.

## 6 USP39 target inhibitor

The catalytic domains of most USP family members are structurally similar to the human right hand, with three substructural domains: finger, palm, and thumb. Between the palm and thumb substructural domains is a catalytic triad of cysteine, histidine, and aspartate catalytic residues, and the catalytic triad is critical for the effective deubiquitination function of USP family members (95, 96). However, replacing the three catalytic residues of USP39 with other amino acid residues causes USP39 to lose its classical deubiquitination activity, which makes the development of inhibitors difficult. Although relevant studies have demonstrated that USP39 still possesses deubiquitination function, we can no longer focus on targeting only the active amino acid residues that bind to the catalytic structural domain for inhibitor site discovery. Since USP39 has the ability to modulate pre-mRNA splicing to affect tumor progression, we propose that compared to other USP inhibitors, such as USP7 inhibitors, which inhibit USP7 deubiquitination function by covalently or non-covalently binding to catalytic residues of the catalytic structural domain (97), USP39 inhibitors may inhibit USP39 deubiquitination activity by binding to the reaction site of the non-catalytic structural domain to inhibit the malignant progression of tumors.

IK is a protein molecule involved in spliceosome assembly and can effectively promote spliceosome activation (98), while USP47 can indirectly promote ATM pre-mRNA splicing by deubiquitinating and stabilizing IK (99). Similarly, USP39 is able to regulate pre-mRNA splicing of specific molecules by binding to the splicing regulators RBM39, SRPK1, SRSF1, and others. In addition, similar to other members of the USP family, USP39 can also directly or indirectly bind to tumor regulatory molecules to prevent degradation of specific proteins by removing the attached ubiquitin chains from their amino acid residues. The main difference is that the site where USP39 contacts specific proteins is not a catalytic structural domain site, but may be a site from a non-catalytic conserved structural domain. Therefore, based on the above idea, future research should focus on exploring the specific mechanism of non-catalytic sites of USP39 and screening for possible target compounds to evaluate its tumor inhibitory effect.

In conclusion, although no effective inhibitors of USP39 have been developed in the clinic, we can still provide a variety of effective research options based on the structure and function of USP39.The development of USP39 inhibitors is certainly novel and innovative compared with those targeting the traditional catalytic site. We will conduct a large number of experiments in the future to verify this conjecture.

## 7 Summary and outlook

**Figure 4:**
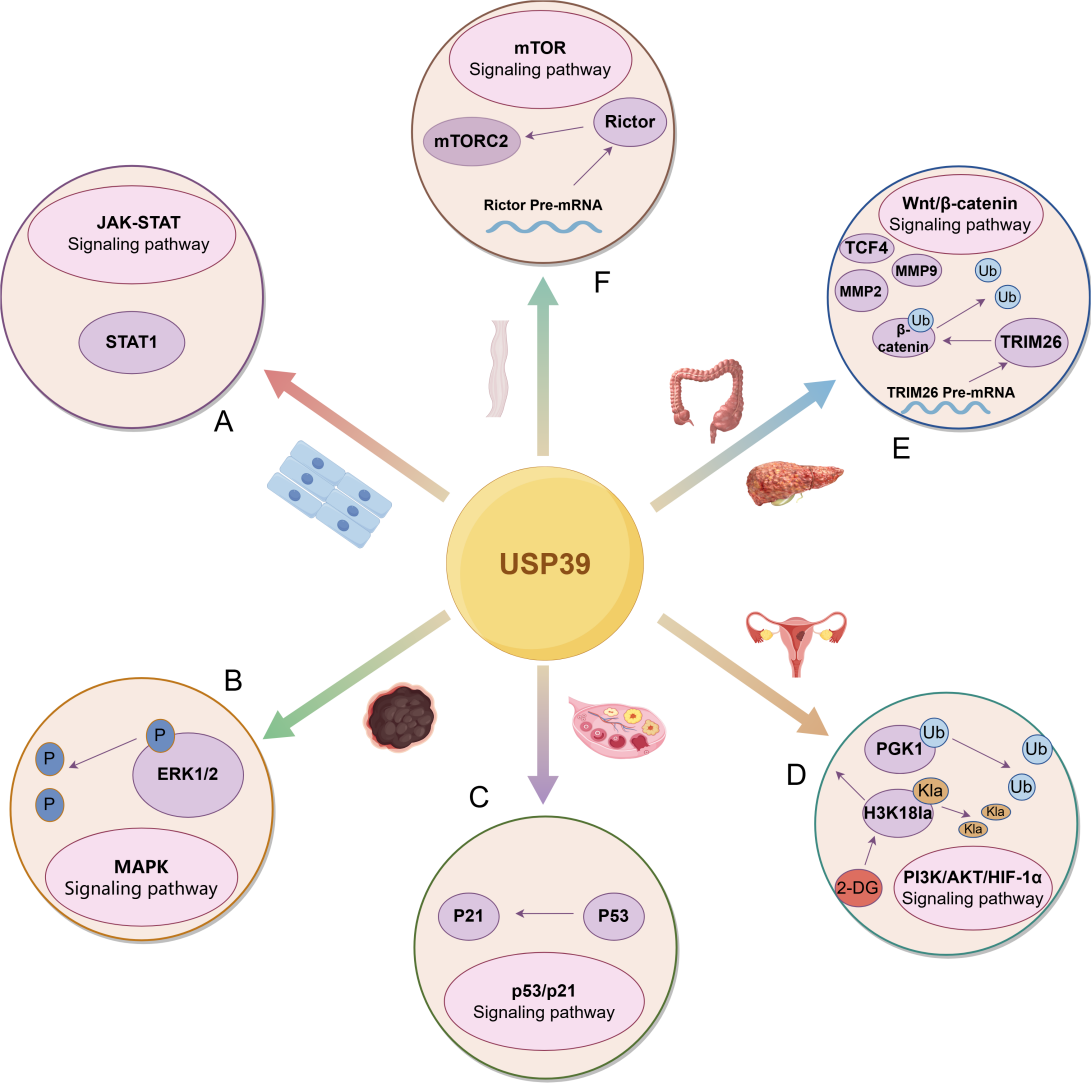
USP39 drives the malignant progression of tumors by regulating multiple signaling pathways. **(A)** In head and neck squamous cell carcinoma, USP39 positively regulates STAT1 expression, enhancing its role in the JAK-STAT signaling pathway. **(B)** In melanoma, USP39 modulates the activation of the MAPK signaling pathway by regulating ERK1/2 phosphorylation levels. **(C)** In ovarian cancer, USP39 regulates p53 expression, thereby modulating the activation of the p53/p21 signaling pathway. **(D)** In endometrial carcinoma, USP39 regulates PGK1 expression by deubiquitinating and stabilizing PGK1. Additionally, H3K18la directly binds to USP39 to influence its expression, a process that can be inhibited by 2-DG. **(E)** USP39 interacts with β-catenin through deubiquitination while also indirectly regulating its expression by modulating the splicing of TRIM26 pre-mRNA. Furthermore, USP39 influences key downstream targets of signaling pathways, including TCF4, MMP2, and MMP9. **(F)** In esophageal squamous cell carcinoma, USP39 regulates the splicing of Rictor pre-mRNA, thereby modulating the activation of mTORC2.The figure was generated using Figdraw. (https://www.figdraw.com/static/index.html#/).

In a word, the research efforts dedicated to USP39 have allowed the discovery of a wide range of USP39’s regulatory roles in the development of malignant tumors (as shown in **Figure 4**). As a key regulator of malignant progression of malignant tumors, USP39 is abnormally overexpressed in the tissues and cells of malignant tumors, and plays a role in modulating diverse biological processes, including tumor cell growth, proliferation, invasion, cell cycle progression, and apoptosis. Therefore, USP39 could reasonably be regarded as a promising biomarker for the early detection and surveillance of tumor progression. In addition, in the study of tumor drug resistance, the overexpression of USP39 can increase the resistance of colorectal and ovarian cancer cells to chemotherapeutic drugs; this resistance can be reversed by knocking down USP39 expression. These findings provide a new theoretical framework and basis for improving clinical chemotherapy strategies. Additionally, in the study of tumor prognosis, it was found that USP39 was closely related to the prognosis of patients with hepatocellular carcinoma, pancreatic ductal adenocarcinoma, glioma, neuroblastoma and other malignant tumors. This suggests its potential as a prognostic biomarker for malignant tumors and offers new insights into their prevention and treatment.

However, current research on USP39 is still limited. Most of the relevant studies on USP39 in malignant tumors have been limited to phenotypic and functional studies, lacking further exploration of mechanisms and pathways. In addition, in the phenotypic studies of head and neck squamous cell carcinoma, leukemia, endometrial carcinoma, and non-small cell lung cancer, the relatively small number of tumor tissue specimens may limit a comprehensive evaluation of USP39 expression levels and its heterogeneity across different tumor types. To improve generalizability and scientific rigor, future studies will need to include a larger sample size. Furthermore, existing studies have only explored the role of USP39 in regulating tumor-related signaling pathways, including PI3K/AKT/HIF-1α and Wnt/β-catenin, across a limited range of cancers, such as hepatocellular carcinoma, endometrial cancer, and ovarian cancer. Future research is required to comprehensively explore the specific mechanisms through which USP39 governs malignant progression across various types of cancers. Moreover, drug resistance studies of USP39 in malignant tumors have not been mentioned in most malignant tumors, but only in a few malignant tumors, such as colorectal cancer, ovarian cancer, etc. The role of USP39 in anticancer drug resistance and the specific mechanism of action still need to be further explored. Furthermore, the development of USP39 inhibitors has not been mentioned in any study, highlighting a significant gap in this field. In this paper, we only predicted the possible binding sites in the protein structure of USP39, but we did not screen out the suitable sites and compounds, which will require extensive experimental validation in the future. The development of USP39 inhibitors by exploring effective targets will be the focus of future research in this field.

In summary, USP39 holds great potential as a clinical biomarker and therapeutic target in oncology, providing novel approaches and pathways for precise cancer detection and effective treatment. Based on the current understanding of USP39’s regulatory mechanisms, further refinement of cancer treatment strategies can be achieved. First, USP39 can influence malignant tumor progression through multiple regulatory mechanisms simultaneously. For example, in HCC, USP39 not only specifically binds to molecules such as SP1 and ZEB1, removing ubiquitin chains to enhance their protein stability and thereby promoting tumor initiation and progression, but also impacts malignant tumor progression by regulating the pre-mRNA splicing of molecules such as AuroraB, FoxM1, and TRIM26. On this basis, the combined use of drugs that inhibit both USP39’s binding functions and splicing regulatory functions can exert multifaceted inhibitory effects, effectively suppress tumor progression and enhance therapeutic efficacy. Furthermore, USP39 can cooperate with multiple oncogenic factors to promote tumorigenesis in malignant tumors. For instance, in HCC, cervical squamous cell carcinoma, and breast cancer, USP39 and FoxM1 exhibit a synergistic effect in driving malignant tumor progression. Therefore, applying dual-targeted or multi-targeted combination therapies based on this mechanism could significantly improve treatment efficacy and increase patient survival rates. Additionally, USP39 can indirectly influence tumor progression by regulating abnormal glycolysis, glutamine metabolism, and tumor angiogenesis. Combining metabolic inhibitors with anti-angiogenic drugs could further enhance treatment outcomes by strengthening the inhibition of tumor metabolic adaptability and angiogenic potential. These novel cancer treatment strategies based on the unique characteristics of USP39 are of great significance for the development of precision medicine. Clinicians can customize personalized treatment strategies according to tumor type, tumor-related molecules, changes in the tumor microenvironment, and individual patient needs, significantly improving therapeutic efficacy while minimizing adverse effects.
